# MicroRNAs in muscle wasting

**DOI:** 10.1002/jcsm.12384

**Published:** 2019-01-29

**Authors:** Tsuyoshi Suzuki, Jochen Springer

**Affiliations:** ^1^ Department of Cardiology and Pneumology University Medical Center Göttingen (UMG) Göttingen Germany

**Keywords:** Muscle wasting, MicroRNA, Cachexia

## Introduction

Skeletal muscle makes up approximately 40% of the total body mass; it is essential in providing structural support, to regulate motion and as an energy store, thereby playing a major role in the overall metabolism. Skeletal muscle retains a high plasticity in order to respond to various stimuli, which subsequently lead to changes in gene transcription and translation. Aside from the obvious transcription factors, non‐coding RNAs have received much attention over the last decade and can be subclassed into long non‐coding RNA and small non‐coding RNA termed microRNA (miR). These miRs are similar to mRNA when first transcribed as primary RNA and are subsequently processed by the endoribonuclease DROSHA associated with PASHA to a precursor miR, which is further processed by the endoribonuclease DICER1 to form mature miRs.^1^ The mature miR binds to its target mRNAs leading to a blocked translation or degradation thereby providing the cell with a post‐transcriptional control of gene expression.^2^, ^3^

While some miRs are expressed ubiquitously in most tissues and cell types, other miRs are highly and specifically enriched in certain tissues.^4^ MyomiRs comprise a group of miRs, who display an enriched expression in skeletal muscle including miR‐1, miR‐133a, miR‐133b, miR‐206, miR‐208, miR‐208b, miR‐486, and miR‐499. These miRs are under the transcriptional control of myogenic regulatory factors such as MyoD, myogenin, Myf5, and MRF4.^5^ The expression of MyomiRs is modulated in skeletal muscle growth, its development and maintenance, and during atrophy.^5^ Two key players of muscle wasting are the E3 ubiquitin ligases MAFbx and MuRF‐1, the latter being the only E3 ubiquitin ligase known to target contractile proteins in catabolic conditions^6^ and which can be inhibited by small molecules.^7^ The related proteins MuRF‐2 and MuRF‐3 bind to microtubules and are implicated in sarcomere formation with evident functional redundancy, which has proven to be important for the maintenance of skeletal muscle, as double knockout mice lead to myopathy, reduced fore generation, and fibre type shift.^8^ In contrast to healthy adaptation, not only myomiRs are regulated in cancer cachexia, a recent publication showed an up‐regulation of hsa‐miR‐3184‐3p, hsa‐miR‐423‐5p, hsa‐let‐7d‐3p, hsa‐miR‐1296‐5p, hsa‐miR‐345‐5p, hsa‐miR‐532‐5p, hsa‐miR‐423‐3p, and hsa‐miR‐199a‐3p, but no down‐regulation of miRs in skeletal muscle biopsies of patients with pancreatic and colorectal cancer (Table 1).^9^ In a rat model of paralysed muscle by spinal cord injury, a down‐regulation of miRs 23a, 23b, 27b, 145, and 206 was observed 56 days after injury,^10^ while injection of 30 μg of mir‐206 attenuated muscle loss in a rat denervation model.^11^ In patients with chronic obstructive pulmonary disease (COPD), an up‐regulation of miR‐542‐3p/5p in quadricep muscle has been described, which caused muscle wasting and reduced mitochondrial function when overexpressed in mice possibly due to a suppression of the mitochondrial ribosomal protein MRPS10, reduced 12S ribosomal RNA expression, and increased TGF‐b signalling.^12^ In patients with COPD with a low fat free mass, an increased expression of miR‐675 in quadricep muscle was shown to repress muscle regeneration *in vitro*.^13^ Moreover, quadricep expression of miR‐422a was positively associated with muscle strength (maximal voluntary contraction *r* = 0.59, *P* < 0.001 and *r* = 0.51, *P* = 0.004, for COPD and aortic surgery, respectively) and inversely associated with the amount of muscle that would be lost in the first post‐operative week (*r* = −0.57, *P* < 0.001).^14^ Overexpression of miR‐23a/27a in muscle attenuated diabetes‐induced muscle cachexia and attenuates renal fibrosis lesions via muscle‐kidney crosstalk in streptozotocin‐induced diabetic mice.^15^ Recently, the lncRNA MAR1 has been shown to act as a miR‐487b scavenger to regulate Wnt5a protein expression leading to stimulated muscle differentiation and regeneration as well as increased strength in mice^16^ making the already complex miR regulatory system even more complicated.

**Table 1:** Differential regulation of miR expression in skeletal muscle after exercise

miR up‐regulated	miR down‐regulated	Exercise type	Exercise duration	Reference
miR‐1, miR‐133a, miR‐133b, miR181a	miR‐9, miR‐23a, miR‐23b, miR‐31	Acute exercise	Acute bout of moderate‐intensity endurance cycling	Russel *et al*.^25^
	miR‐1, miR‐133a	Acute resistance exercise	45 min of one‐legged knee extensor exercise	Ringholm *et al*.^26^
	miR‐1	12 weeks of training with two weekly resistance exercise sessions	12 weeks of training with two weekly resistance exercise sessions	Mueller *et al*.^27^
	miR‐1, miR‐133a, miR‐133b, miR‐206	Endurance	Cycle ergometer five times per week frequency for 12 weeks	Nielsen *et al*.^28^
miR‐1, miR‐29b		Endurance	10 days of endurance training	Russel *et al*.^25^
miR‐136, miR‐200c, miR‐376, miR‐377, miR‐499b, miR‐558	miR‐28, miR‐30d, miR‐204, miR‐330, miR‐345, miR‐375, miR‐449c, miR‐483, miR‐509, miR‐520a, miR‐548, miR‐628, miR‐653, miR‐670, miR‐889, miR‐1245a, miR‐1270, miR‐1280, miR‐1322, miR‐3180	Chronic resistance exercise	12‐week lower body resistance exercise	Ogasawara *et al*.^29^
miR‐451	miR‐26a, miR‐29a, miR‐378	Resistance exercise	12‐week resistance exercise training program (pushing, pulling, and leg exercises, with 60 weight‐lifting sessions in total	Davidsen *et al*.^30^
	miR‐133a, miR‐378, miR‐486	Resistance exercise	8 × 5 unilateral leg press repetitions on each leg at 80% of the 1repitition maximum	Fyfe *et al*.^31^

miRs can be actively secreted from a cell or leaking through the membrane in response to various stimuli and insults resulting in varying circulating miR levels in the blood, which are relatively stable making miRs interesting for the use as biomarkers and therapeutic targets.^1^ This is of particular importance in muscle wasting, as there are very few blood‐based biomarkers such as myostatin or agrinin that correlated with muscle mass.^17^, ^18^, ^19^, ^20^, ^21^ Several other circulating factors like GDF‐15,^22^ activin A,^23^ and low testosterone^24^ have been associated with muscle loss and survival in sarcopenia and cachexia and therefore can be considered potential biomarkers, but need to be validated in large trials. miRs could serve not only as biomarkers for muscle status and wasting but also as biomarkers to monitor muscle regeneration and therapy effects.

Resistance exercise has been of particular interest in sarcopenia and also in cachexia.^32^, ^33^, ^34^, ^35^, ^36^, ^37^ Moreover, exercise mimetics such as trimetazidine are of interest in the therapy of muscle atrophy,^38^ but also need companion biomarkers. MyomiRs are strongly regulated in resistance exercise, and their expression patterns in muscle as well as their plasma pattern levels may have the potential to serve as biomarkers for exercise, and regular monitoring in sarcopenic or cachectic patients could prevent detrimental over‐exercise.

## Conflict of interest

None declared.
